# Characterization of change in the Harike wetland, a Ramsar site in India, using landsat satellite data

**DOI:** 10.1186/2193-1801-3-576

**Published:** 2014-10-01

**Authors:** Samson Okongo Mabwoga, Ashwani Kumar Thukral

**Affiliations:** Department of Botanical and Environmental Sciences, Guru Nanak Dev University, Amritsar, 143 005 Punjab India; School of Tourism and Natural Resources Management, Department of Environment, Forestry and Agriculture, Maasai Mara University, P.O. Box 861-20500, Narok, Kenya

**Keywords:** Change detection, Post-classification, Punjab, Remote sensing, Unsupervised classification

## Abstract

The increasing population in the developing countries has rendered wetlands vulnerable to land use changes. Remote sensing offers a rapid and efficient means of data acquisition of ecosystems in time and space. The present study was undertaken to identify changes in the Harike wetland, a Ramsar site in the state of Punjab, India; and identify causal factors, as well as vulnerable areas threatened from the land cover changes. Unsupervised classification and post-classification change detection techniques were applied to Landsat Thematic Mapper (TM) and Enhanced Thematic Mapper Plus (ETM+) data of 16-10-1989, 22-10-2000 and 26-10-2010. Images were classified into five land cover classes (1) Waterbody, (2) Wetland I, (3) Wetland II, (4) Barren land and (5) Agricultural land. Land cover change is characterized mainly by a decrease in the wetland area, as indicated by decrease in wetland vegetation and an increase in non-wetland areas, characterized by increasing agricultural and barren land areas. Overall, the wetland shrunk by 13% from 1989 to 2010, with the north-eastern side experiencing maximum shrinkage. The wetland needs immediate reclamation to check it from further shrinkage so as to save its biodiversity.

## Introduction

Despite the ecological importance of wetlands, intense anthropogenic pressure is diverting these landscapes to agriculture and habitation use. The extent of ecological changes in wetlands in many parts of the world has been increasing in recent years (Finlayson 1994). Changes may be due to a combination of both natural and anthropogenic factors (Han et al. 2007; Xie et al. 2010), but human-induced changes are usually more rapid than the natural ones. Recently, wetland changes are experiencing a shift towards anthropogenic land cover types, suggesting an increase in human activity (Gibbes et al. 2009). In combination with other factors, humans cause direct wetland losses mainly through agriculture (Syphard and Garcia 2001). With advancements in computing, the use of remote sensing (RS) and geographical information systems (GIS) has made it feasible to study wetlands in time and space.

Wetland changes can be studied using RS data and a combination of different components that result from such changes (Melendez-Pastor et al. 2009). Landsat Multispectral Sensor (MSS), TM and ETM + have been extensively used in wetland studies, for spectral discrimination of wetland ecosystems (Ozesmi and Bauer 2002). Landsat data have proved to be useful for change detection in many studies on wetlands (Ramsey and Laine 1997; Munyati 2000; Harvey and Hill 2001; Nelson et al. 2002; Chen et al. 2003; Pavri and Aber 2004; Baker et al. 2007; Kiage et al 2007; Ma et al. 2007; Owor et al. 2007; Tagil 2007; Carreno et al. 2008).

Change detection process identifies differences in the state of an object or phenomenon by observing it at different times (Singh, 1989). Some of the most common methods used for change detection include image differencing, principal component analysis and post-classification comparison (Lu et al. 2004). Post-classification change detection has been found to be the least sensitive to changes in the image properties of class separability, radiometric normalization error and band correlation, and is conceptually one of the simplest change detection methods which involves an initial, independent classification of each image, followed by a thematic overlay of the classifications resulting in a complete “from-to” change matrix of the transitions between each class on the two dates (Almutairi and Warner 2010).

### Study area

**Figure 1:**
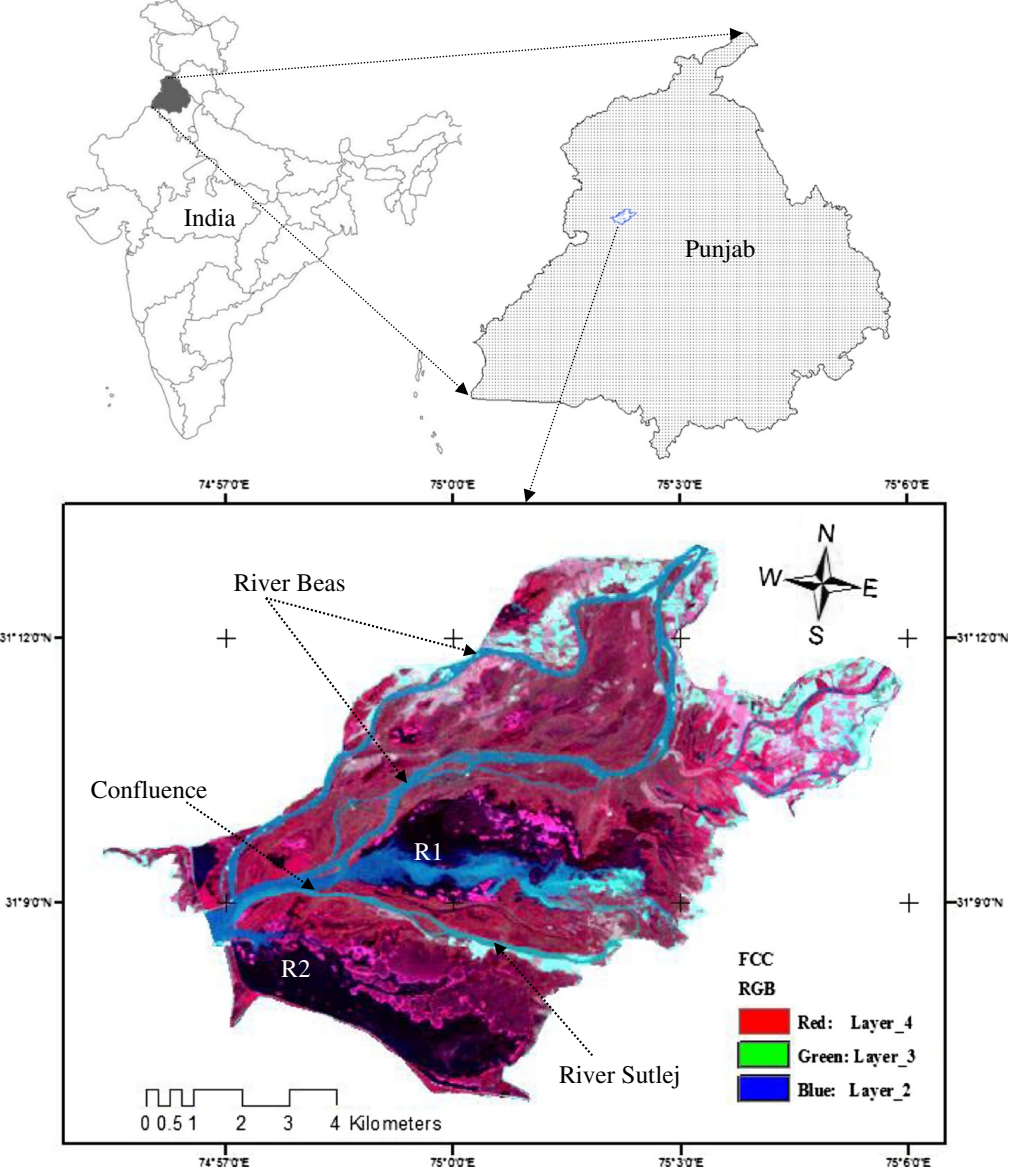
**Geographical location of the study area.** R1 and R2 = River Beas and Sutlej reservoirs respectively.

The present study was carried out on Harike wetland, an internationally recognised Ramsar site covering an area of 8739 ha (Table 1). The wetland falls between 31°08′N to 31°23′N latitudes and 74°90′E to 75°12′E longitudes (Figure 1). It is surrounded at its periphery by agricultural land on all sides and is located at the confluence of the rivers Beas and Sutlej and was formed when a barrage was constructed in the year 1952 with the aim of storing and providing irrigation and drinking water to parts of the Southern Punjab and to the adjoining State of Rajasthan. It is significant for ecological, economic, scientific, socio-cultural as well as for recreational purposes. Harike wetland also supports rare, vulnerable and endangered plants, fish and other faunal species and attracts large populations of avifauna during the winters, from places as far off as Siberia (Ladhar 2002). In the recent times, rare fresh water dolphins (*Platanista gangetica minor*) were sighted in the wetland (The Hindu The Hindu 2008).

**Table 1:** **Extent and relative land cover area (in ha) for the years 1989, 2000 and 2010 and their percentages**

Land Cover class	Land cover extent					Relative change						
	1989	%	2000	%	2010	%	1989- 2000	%	2000-2010	%	1989-2010	%
	Area		Area		Area		Area		Area		Area	
	(ha)		(ha)		(ha)		(ha)		(ha)		(ha)	
Waterbody	2414	28	1566	18	2026	23	-848	-35	459	29	-388	-16
Wetland I	745	9	404	5	468	5	-340	-46	63	16	-277	-37
Wetland II	3995	46	4224	48	3527	40	229	6	-698	-17	-469	-12
Barren land	796	9	1218	14	1190	14	421	53	-28	-2	394	49
Agricultural land	789	9	1326	15	1528	17	537	68	202	15	739	94
Total	8739	100	8739	100	8739	100

Harike wetland has been recognized as an important waterfowl habitat at various platforms, especially by the Ministry of Environment and Forests (MoEF), Government of India, declaring it a wildlife sanctuary in 1982. It was also identified as a site for conservation under the Indian National Wetlands Programme (1987-1988) and included in its conservation and management programmes. In 1990, Harike wetland was designated as a Ramsar site (Ramsar Convention 2008). At the State level it was declared a bird sanctuary (Harike Wetland Bird Sanctuary) by the State government of Punjab in 1992, though fishing was allowed. However, in the year 2000 when it came under the Wildlife Act, fishing was totally banned in the wetland. Currently it is under the administration of the State government, but the MoEF has overall responsibility for administering and enforcing environmental laws and policies affecting the wetland. The State government undertakes various activities for conservation of the wetland including afforestation, protection of wildlife, control of weeds such as water hyacinth, conservation of soils and water quality.

Indian wetlands are facing increasing anthropogenic pressure from development and population growth, agriculture, deforestation, alternation of hydrology through drainage and over-extraction of water for irrigation (Prasad et al. 2002). In the Punjab, most of the wetlands are undergoing general ecological degradation and the attitude of the public is minimal with respect to ecological restoration (Ladhar 2002). This has made the wetlands in thePunjab to lose their natural aquatic character. Out of the several wetlands in the State of Punjab, Harike, Kanjli, Ropar, Dholbaha, Januari and Ranjit Sagar are some of the important wetlands. Some wetlands in the State have lost their ecological integrity and have become extinct. This is due to the drainage into agricultural cropland, discharge of domestic and industrial effluents, erosion from surrounding catchments resulting in siltation and invasion by alien weeds particularly the water hyacinth (Ladhar 2002). A combination of both natural and anthropogenic factors has been reported as the causal factors of wetland change in the Harike wetland area (Chopra et al. 2001; Ladhar 2002). However, no efforts have been made to quantify the changes in the wetland. The objectives of this study were to quantify changes in the land cover of the wetland between the years 1989 to 2010, identify causal factors, threats and vulnerable areas threatened due to these land cover changes.

## Materials and methods

### Image pre-processing

Satellite images of Landsat TM and ETM + scenes covering the study area were obtained from the USGS (http://glovis.usgs.gov/). The image characteristics for TM and ETM + sensors are given in Table 2. The multi-temporal Landsat images acquired on 16 -10 -1989, 22-10-2000 and 26-10-2010 had 0% cloud cover. All image processing was performed using ERDAS Imagine 9.1, and ArcView GIS 3.2. Wetland maps were prepared in ERDAS imagine and Surfer 8. An eTrex, Garmin Global Positioning System (GPS) receiver was used to determine the geo-coordinates of a given area in terms of its latitude and longitude.

**Table 2:** **Image characteristics of the landsat TM and ETM+ sensors**

	Resolution					
Sensor/Path/Row	Spectral		Spatial (m)	Temporal (Days)	Radio-metric	Swath (km)
	Bands	Wavelength (μm)				
LANDSAT	Band 1 (Blue)	0.45 – 0.52	30		8 bit	
TM/148/038	Band 2 (Green)	0.52 – 0.60	30			
	Band 3 (Red)	0.63 – 0.69	30			
	Band 4 (NIR)	0.76 – 0.90	30	16		185
	Band 5 (Mid NIR)	1.55 – 1.75	30			
	Band 6 (Thermal)	10.4 – 12.5	120			
	Band 7 (Mid NIR)	2.08 – 2.35	30			
LANDSAT	Band 1 (Blue)	0.450 – 0.515	30		8 bit	
ETM+/148/038	Band 2 (Green)	0.525 – 0.605	30			
	Band 3 (Red)	0.630 – 0.690	30			
	Band 4 (NIR)	0.760 – 0.900	30	16		185
	Band 5 (Mid NIR)	1.550 – 1.750	30			
	Band 6 (Thermal)	10.400 – 12.5	60			
	Band 7 (Mid NIR)	2.080 – 2.35	30			
	Band 8 (PAN)	0.520 – 0.92	15			

### Satellite image processing

The Landsat images used in this study have been orthorectified to remove distortion due to topographic variation. The images were subset to limit image processing to the area of interest (AOI), to make image processing easier and to extract the wetland area. Prior to sub-setting, a reconnaissance survey was done in the wetland area to develop baseline information on the extents of the whole wetland. A base map was prepared for the wetland area using ERDAS Imagine 9.1 and ArcView GIS (Figure 1).

Since TC and PCA is calculated from reflectance, the data were converted to reflectance, a physical measurement, by converting to radiance and then to reflectance, using the latest gain and bias numbers, and the LMIN_λ,_ LMAX_λ,_ for the Landsat 5 TM and Landsat 7 ETM + sensors given in Chander et al. ( 2009). The graphical model in ERDAS was used for the conversion of DN values to reflectance. The images were also atmospherically corrected using the dark object subtraction (DOS) method.

### Image transformations

To decrease both information redundancy and the number of layers used for the unsupervised classification (Frankovich, 1999), two image transformations were used; principal components analysis (PCA) and tasseled cap (TC) transformation. PCA was applied to the multi-temporal Landsat TM and ETM + image data of 16-10-1989, 22-10-2000 and 26-10-2010. Two PCAs were applied separately to Landsat TM/ETM+; (i) on the visible bands 1, 2 and 3 and (ii) on the mid-infrared bands 5 and 7, since they are highly correlated. The infra-red band 4 was left untouched as it is less correlated to either the visible bands or the mid-infra-red band. This reduced the number of layers from six to three.

Another transformation used was Tasseled Cap Transformation (TC). Using dataset from Landsat 5 TM (1989), Landsat 7 ETM + (2000) and Landsat 5 TM (2010) for the Harike wetland, TC-transformed images were produced with the help of ERDAS. The transformation was performed on six TM/ETM + bands and six new layers were produced. Two of the six tasseled cap transform bands were used as the first two of these contain most of the information; Band 1 (brightness, measure of soil) - soil brightness index (SBI), Band 2 (greenness, measure of vegetation) - green vegetation index (GVI). The use of SBI and GVI layers along with band 4 help to separate vegetation from bare features during the classification process.

The first PCAs (that of bands 1, 2 and 3, and bands 5 and 7), the infra-red band 4, and soil brightness index (SBI) and the green vegetation index (GVI) from tasseled cap transformation were merged to form a five layer image and was utilized for unsupervised classification. The preprocessed and spectrally enhanced images (using PCA, and TC transformation), for the dates 16-10-1989, 22-10-2000 and 26-10-2010 were used in image classification. Each image was classified separately using the unsupervised classification method in ERDAS Imagine 9.1 software. Changes in land cover/land use were evaluated using the post-classification comparison method for detection of change.

### Image classification

**Figure 2:**
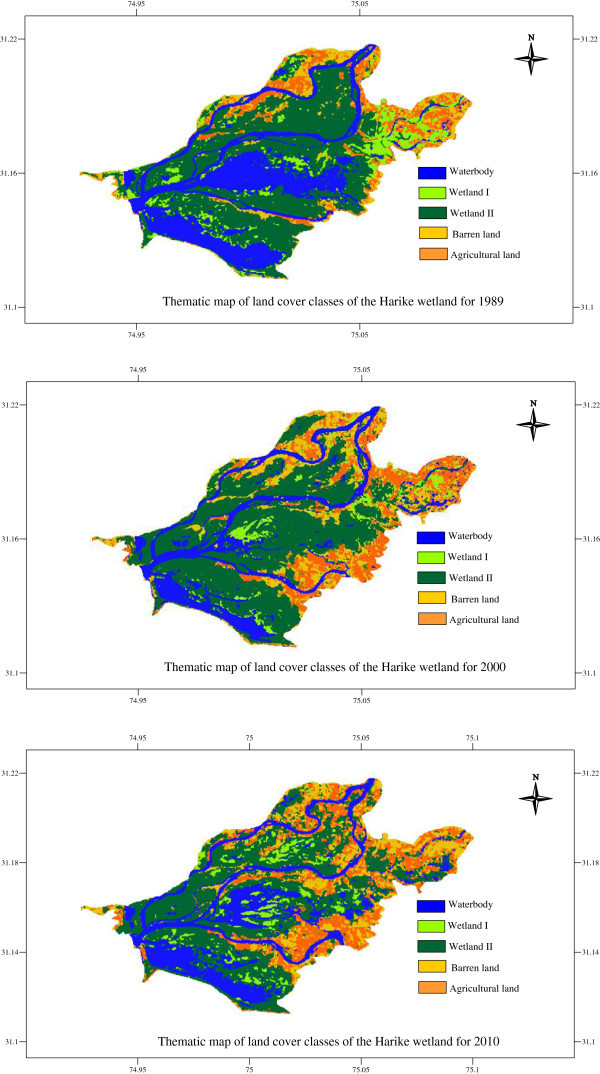
**Landcover classes for Harike wetland between 1989 and 2010.**

Image classification was started with the image dated 26-10-2010, followed by other image dates since this image contained the most recent information to authenticate the classification. From the initially clustered classes, the classes were recoded to simplify interpretation of change. Because of the familiarity of the study area, and using visual inspection of the original images, there was knowledge of the expected classes on the basis of which they were identified. Using the existing knowledge of the study area, 5 classes were identified. The definitions of the final classes is given in Table 3. Classification maps were generated for the images dated 6-10-1989, 22-10-2000 and 26-10-2010 and results of the image classification and thematic maps are given in Figure 2.

**Table 3:** **Wetland cover types used in image classification and interpretation with their definitions**

Class	Definition
1. Waterbody	Areas of open water and small ponds including river Beas and Sutlej and the two reservoirs (Figure 1 - R1 and R2).
2. Wetland I	Dense vigorous vegetation including *Eichhornia*, some grasses and shrubs.
3. Wetland II	Mainly composed of grasses (such as *Typha* and *Arundo* which are the two dominant vegetation types in the wetlands), and woodland vegetation.
4. Barren land	Residential areas, barren land, sandy lands along river Beas and Sutlej and bare soil.
5. Agriculture land	Crop fields and fallow land.

The classified images were compared with GPS, topographical sheets and available wetland maps of the study area, to determine how each site represented on the ground as observed during ground truthing was classified on the image. Error matrices as described by Congalton and Green ( 1999) were used for assessing the accuracies. The overall accuracies for the classifications were calculated to be 74.84%, 81.65% and 81.41% respectively for 1989, 2000 and 2010 images.

## Results and discussion

### Image classification and change detection

The image classification of the wetland resulted into five land cover types: Waterbody, Wetland I, Wetland II, Barren land and Agricultural areas (Table 1). Analysis of the Landsat TM and ETM + data provided land cover types and their respective changes within the study period examined.

Surface water accounting for 28% (2414 ha), 18% (1566 ha) and 23% (2026 ha) of the wetland area, and Wetland II 46% (3995 ha), 48% (4224 ha) and 40% (3527 ha) for the years 1989, 2000 and 2010 respectively formed the largest classes in the wetland. There was a decrease in the Wetland I vegetation from 1989 (9% - 796 ha) to 5% (404 ha) in 2000 and 2010. Barren area increased by 5% (434 ha) from 1989 to 2000 and the area did not change significantly in 2000 and 2010, but remained constant at 14% (1218 and 1190 ha respectively). However, Agricultural land increased by 6% (545 ha) from 1989 to 2000 and then by 2% (204 ha) between 2000 and 2010. Changes were prominent on the north eastern side of the wetland and corresponded to an increased agricultural land.

The dated 16-10-1989 image was used as the base year for change detection and the changes occurring until 2010 were determined. Change detection was conducted for two time periods: a) 1989 to 2000, and b) 2000 to 2010. In the first period of 11 years between 1989 and 2000, the area under Waterbody area decreased by 9.7% (848 ha). It increased during the next 10 years (between 2000 and 2010) by 5.3% (459 ha). There was a decline in Wetland I vegetation by 3.9% (340 ha) in the first period and an increase by 0.7% (63 ha) in the second, whereas Wetland II exhibited an increase by 2.6% (229 ha) and a subsequent decrease by 7.8% (698 ha). Barren land areas increased by 4.8% (421 ha) in the first period followed by a decrease by 0.3% (28 ha) during the second period. Agricultural land increased during the two periods by 6.1% (537 ha) and 2.3% (202 ha) respectively.

There were no consistent changes observed during these two periods. The decrease in Wetland I could have resulted due to the efforts by the State government, with a task assigned to the Indian Army, in an operation launched in 1999 to rid the wetland of the menace of water hyacinth, which culminated in removal of 0.15 million tons of the weed. From the change matrix (Table 4c), it could be observed that 8% (62 ha) of Wetland I converted to Waterbody. Afforestation efforts were also on from the 1990s after the wetland was declared a bird sanctuary, which could have caused the increase in area of Wetland II category towards 2000. Observations during the 21 year period between 1989 and 2010 showed that the Waterbody area decreased by 4.4% (388 ha), Wetland I by 3.2% (277 ha) and Wetland II by 5.4% (469 ha) whereas Barren and Agricultural land areas increased by 4.5% (394 ha) and 8.5% (739 ha) respectively. Overall, the wetland had shrunk by 13% from 1989 to 2010. The loss of wetland between 1989 and 2000 was greater than between 2000 and 2010. During the first study period of 11 years the wetland area was reduced by 11%, followed by 2% decrease in the next 10 years.

**Table 4:** Change matrix* showing the total area (in ha) converted from one land cover to the next during 1989 - 2010

a						
1989-2000	Waterbody	Wetland I	Wetland II	Barren land	Agricultural land	2000 Total
Waterbody	**1137**	62	245	64	59	1566
Wetland I	150	**49**	120	32	53	404
Wetland II	922	406	**2724**	101	71	4224
Barren land	101	105	450	**283**	279	1218
Agricultural land	104	122	456	317	**327**	1326
1986 Total	**2414**	**745**	**3995**	**796**	**789**	**8739**
**b**						
**2000-2010**	**Waterbody**	**Wetland I**	**Wetland II**	**Barren land**	**Agricultural land**	**2010 Total**
Waterbody	**1110**	139	631	81	65	2026
Wetland I	32	**20**	393	12	10	468
Wetland II	272	109	**2682**	291	172	3526
Barren land	51	72	228	**369**	470	1190
Agricultural land	102	64	290	463	**611**	1529
2000 Total	**1567**	**404**	**4224**	**1215**	**1329**	**8740**
**c**						
**1989-2010**	**Waterbody**	**Wetland I**	**Wetland II**	**Barren land**	**Agricultural land**	**2010 Total**
Waterbody	**1367**	108	420	68	62	2026
Wetland I	157	**48**	245	7	10	468
Wetland II	601	361	**2241**	165	158	3527
Barren land	82	107	447	**275**	279	1190
Agricultural land	206	120	642	279	**281**	1528
1986 Total	**2414**	**744**	**3995**	**795**	**790**	**8738**

*Unchanged areas are in the diagonals (in bold) from top left to bottom right of the matrix.

**Figure 3:**
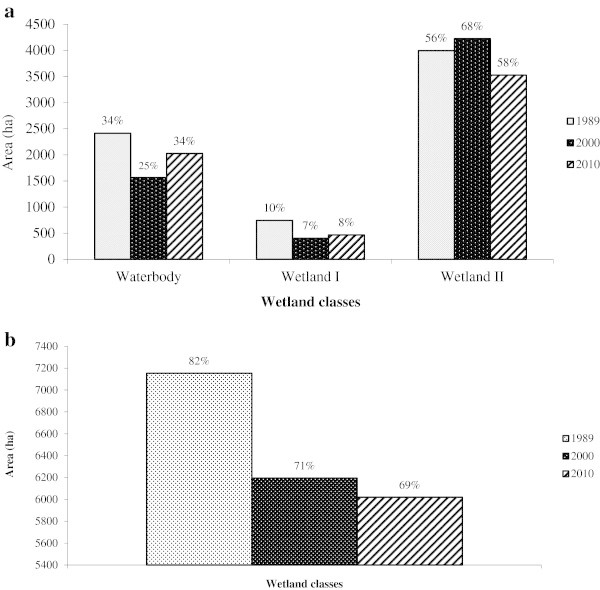
**Changes in true wetland classes for the Harike wetland. a**. Changes in true wetland classes between 1989 and 2010; **b**. Overall changes in wetland classes between 1989 and 2010.

Out of the 5 wetland classes, separate analyses were made for Waterbody, Wetland I and Wetland II that formed wetland classes (Table 5) and Barren land and Agricultural land areas, which constituted non-wetland classes (Table 5). Bar graphs of these classes are shown in Figure 3a and b. Wetland II accounted for the largest area constituting 56% (3995 ha) in 1989, 68% (4224 ha) in 2000, and 59% (3527 ha) in 2010 of the wetland areas, followed by Waterbody area with 34% (2414 ha), 25% (1566 ha) and 34% (2026 ha) in the respective years, whereas Wetland I occupied 10% (745 ha), 7% (404 ha) and 8% (468 ha) of the area.

**Table 5:** **Area (ha) of true wetland cover classes between 1989 and 2010**

Land cover class	1989		2000		2010	
	Area	%	Area	%	Area	%
	(ha)		(ha)		(ha)	
Waterbody	2414	33.75	1566	25.28	2026	33.65
Wetland I	745	10.41	404	6.53	468	7.77
Wetland II	3995	55.85	4224	68.19	3527	58.58
Total wetland classes	7154	100.00	6195	100.00	6020	100.00
Percentage out of wetland area	82		71		69

**Figure 4:**
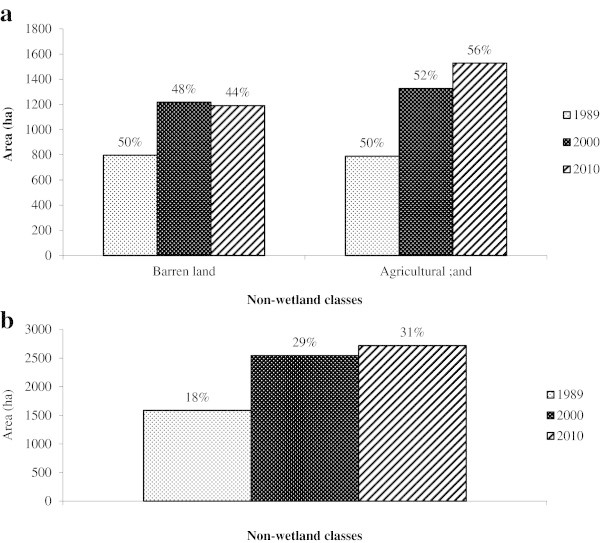
**Changes in non-wetland classes for the Harike wetland. a**. Changes in non-wetland classes; **b**. overall change in the non-wetland classes between 1989 and 2010.

**Figure 5:**
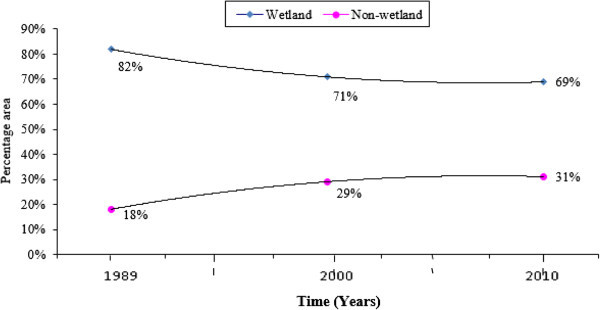
**Trend of changes in area in the Harike wetland.**

To examine how the wetland changed during the study period, these classes were combined and the results revealed (Figure 3b) that the wetland classes decreased from 82% (7154 ha) in 1989 to 71% (6195 ha) in 2000 and gradually to 69% (6020 ha) in 2010 indicating that the wetland area is shrinking. Thirteen percent of the wetland area was lost between 1989 and 2010. Barren land and Agricultural land were non-wetland classes and showed an upward trend with a combined area of 18% (1585 ha) in 1989, increasing to 29% (2544 ha) in 2000, and subsequently to 31% (2718 ha) in 2010 (Table 6 and Figure 4a and b). The trend in changes during the 21 year period of the study in wetland area between the two categories of classes is shown in Figure 5. Wetland showed a declining trend as opposed to the non-wetland.

**Table 6:** **Area (ha) of non-wetland classes between 1989 and 2010**

Land cover class	1989		2000		2010	
	Area	%	Area	%	Area	%
	(ha)		(ha)		(ha)	
Barren land	796	50	1218	48	1190	44
Agricultural land	789	50	1326	52	1528	56
Total non-wetland classes	1585	100	2544	100	2718	100
% out of total wetland area	18		29		31

When performing post-classification change detection, by using the change matrix, an advantage of ‘from-to-’ changes can be taken, to interpret the change information. The land cover extent and change matrices for 1989-2000, 2000-2010 and 1989-2010 are given in Table 4a, b and c. The results provided the direction of change and the extent of different land cover types. Cross tabulation of the land cover area changes showed that of the 388 ha of the Waterbody area lost between 1989 and 2010, 24.9% was converted to the emergent vegetation (Wetland II), 6.5% to Wetland I and the rest to Barren and Agricultural land. Out of the 277 ha of Wetland I area lost from 1989 to 2010, most of it (48.5%) was converted to Wetland II, and 14.5% came under Waterbody area. For Wetland II, of the lost area (469 ha), 16% was converted to Agricultural land and 11% became barren. Wetland areas are among the most sought after for agricultural cultivation.

Clear patterns that highlighted changes to Agricultural land emerged in the classified images as rectangular patches in the image depicting the wetland. These patterns were more prominent on the north-eastern part of the wetland. During the same period, most of the land that was Barren areas was converted to Agricultural land. Strips of Agricultural land also appeared in the interior of the wetland along the River Sutlej, towards the confluence. Classification errors may have also caused unusual changes such as Agricultural land converting to Waterbody. Change matrix showed that 62 ha came under Agricultural land. However, it is likely that the Agricultural land was flooded with Waterbody, to make the land ready for cultivation.

The most prominent conversions observed were: Wetland I to Wetland II, Barren land to Agricultural land and Agricultural land to Barren land. This shows a close interaction between these classes. For example a change from Barren land to Agricultural land indicates that Barren land was put into Agricultural land in the next time period, and a conversion from Wetland I to Wetland II could mean that marsh areas that were infested with weeds like *Eichhornia* were becoming dominated by emergent vegetation. Other conversions such as from Wetland II to Agricultural land, as well as from Barren land to Agricultural land were an indication of the agricultural expansion. However, it was occurring at the expense of wetland vegetation. It is possible that the wetland areas were cultivated during one planting season and left fallow in another season.

## Conclusion

**Figure 6:**
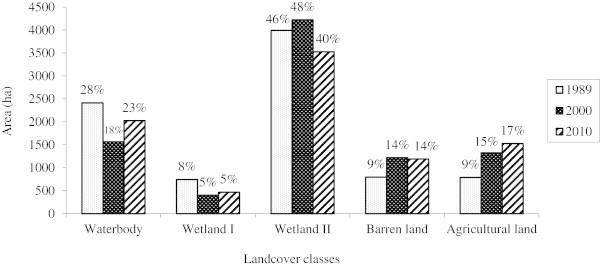
**Land cover (in ha) between 1989 and 2010.**

Widespread anthropogenic impacts on wetlands due to economic and population pressures, and environmental degradation are causing unprecedented changes in the fragile habitats. Undoubtedly, Harike is an important wetland and its characterization is important for the current and future conservation efforts. The destruction of wetland vegetation in the Harike wetland to expand land for Agricultural land, a decrease in the wetland area due to conversion to agricultural land, and an accelerated change in the course of the River Sutlej caused by development of Agricultural land along the river were observed. The wetland is under stress from Agricultural land on the north-eastern side as depicted in the image classification.Land use patterns were analyzed for the years 1989, 2000 and 2010 and in general, Wetland II which is dominated by the grasslands occupied the highest percentage of wetland vegetation, followed by Wetland I. Waterbody area decreased in 2000 and then increased in 2010, whereas wetland II showed a decreasing trend towards 2010. Barren land and agricultural land areas showed an increasing trend from 1989 to 2010. Land cover changes were mainly characterized by a decrease in the wetland area, an increase in agricultural land and Barren areas around the wetland, and inter-conversions between wetland classes. The analysis showed evident shrinkage in the area of the wetland with the major wetland vegetation decreasing. The land cover proportions for the different years are shown in Figure 6.

The study at the Harike wetland unequivocally proved that over the past 21 years, the wetland shrunk by 13% and the area has been lost to Agricultural land and converted to barren areas. The changes observed can be attributed to several factors. It was shown that a part of the wetland has been converted to Agricultural land and Barren land resulting in the reduction of wetland vegetation. This was highlighted from the post-classification change detection along with the change matrices used to identify areas and directions of land cover changes. Comparisons showed that the wetland has changed significantly since 1989 with a retreat in the wetland boundary. Changing water levels between the two periods may have been partly a seasonal phenomenon. Decreasing barren areas towards 2010 and increase in Agricultural land suggest that population pressure is playing a key role in altering the wetland.

It is known that human interference causes significant wetland degradation. A range of human impacts which include drainage, tilling for crop production, grazing, water pollution, stream channelization and dredging affect the stability of wetlands. The increased farming activities around the Harike wetland will affect adjacent wetland areas adversely. Clearance of wetland vegetation by farmers will replace the deep rooted wetland vegetation with shallow rooted crops, which have less soil binding properties than the original vegetation, could lead to increased runoff. While there was a decrease in the Harike wetland area, land cover change data showed that large tracts of grasslands still remain. This creates an opportunity for the management authorities to take appropriate measures to curb further degradation. Agricultural activities adjacent to the rivers Beas and Sutlej not only result in reduction of the wetland size, but also in the deposition of pesticides and fertilizers to have long term impacts on the wetland habitat. Loss of vegetation will also result in loss of habitat, leading to decline in biodiversity in the wetland. The Lake flora and fauna are at risk due to pollution of the lake upstream in the River Sutlej. The present study therefore establishes urgency for saving the Harike wetland from further damage and deterioration.
